# Tumor Molecular and Microenvironment Characteristics in EBV-Associated Malignancies as Potential Therapeutic Targets: Focus on Gastric Cancer

**DOI:** 10.3390/cimb44110390

**Published:** 2022-11-18

**Authors:** Aviva Atri-Schuller, Hassan Abushukair, Ludimila Cavalcante, Stijn Hentzen, Azhar Saeed, Anwaar Saeed

**Affiliations:** 1 Department of Internal Medicine, University of Cincinnati, Cincinnati, OH 45219, USA; 2 Faculty of Medicine, Jordan University of Science and Technology, Irbid 22110, Jordan; 3 Department of Hematology and Medical Oncology, Winship Cancer Institute of Emory University, Atlanta, GA 30322, USA; 4 Department of Internal Medicine, Kansas University Medical Center, Kansas City, KS 66160, USA; 5 Department of Pathology, Huntsman Cancer Institute, University of Utah, Salt Lake City, UT 84112, USA; 6 Department of Medicine, Division of Medical Oncology, University of Kansas Cancer Center, 2330 Shawnee Mission Pkwy, Kansas City, KS 66205, USA

**Keywords:** EBV, EBV malignancies, EBV-associated gastric cancer (EBVaGC), tumor microenvironment (TME), *IFN*-gamma, IDO1, *H. pylori*, TAMs, M1, M2

## Abstract

Although most people are infected with Epstein-Barr Virus (EBV) during their lifetime, only a minority of them develop an EBV-associated malignancy. EBV acts in both direct and indirect ways to transform infected cells into tumor cells. There are multiple ways in which the EBV, host, and tumor environment interact to promote malignant transformation. This paper focuses on some of the mechanisms that EBV uses to transform the tumor microenvironment (TME) of EBV-associated gastric cancer (EBVaGC) for its benefit, including overexpression of Indoleamine 2,3-Dioxygenase 1 (IDO1), synergism between *H. pylori* and EBV co-infection, and M1 to M2 switch. In this review, we expand on different modalities and combinatorial approaches to therapeutically target this mechanism.

## 1. Introduction

Globally, more than 90% of the adult population is estimated to be chronically infected with EBV [1]. Primary infection usually takes place during infancy or adolescence, and it may result in infectious mononucleosis (IM) or it can be asymptomatic. Once primary infection has taken place, the virus becomes latent, persisting in the DNA of the host for the rest of their lifetime, where it may result in cancer development.

EBV is an oncogenic virus; the global burden of EBV-associated malignancies is 1.5%, and the virus is responsible for 1.8% of cancer-related deaths [2]. EBV plays a role in the pathogenesis of multiple lymphoid and epithelial cancers, including Burkitt Lymphoma (BL), Hodgkin’s Lymphoma (HL), NK/T Cell Lymphoma (NKTCL), Nasopharyngeal Carcinoma (NPC), EBV Related Breast Cancer (EBVrBCa), and EBV-Associated Gastric Cancer (EBVaGC) [3]. The oncogenic properties of EBV have been thoroughly studied, with the hopes that by elucidating them, we can develop therapeutic strategies to target them. 

One of these oncogenic properties is EBV’s potential to transform infected cells into cancer cells by modulating the TME of the host for its own viral advantage, which makes the TME a very important contributor to the pathogenesis of EBV-associated malignancies. In fact, the influence of EBV on the TME is strongly associated with the prognosis of EBV-associated malignancies [4]. 

In this review we will focus on specific mechanisms that EBV uses to modulate the TME to its advantage. We will also dive into potential treatments to specifically target the TME, with the hope of expanding the repertoire of therapies for EBV-related malignancies.

## 2. Viruses as Carcinogens

There are multiple mechanisms by which an agent can become carcinogenic. Most often, the DNA replication and repair processes become damaged, and the accumulation of genetic mutations results in cancer [5]. Outside of the DNA sequence, epigenetic modifications that disturb gene expression can also be oncogenic [6]. 

Tumor viruses are associated with 10–12% of cancer cases worldwide [7]. EBV was discovered in the 1960s as the first oncogenic virus. Since then, the International Agency for Research on Cancer (IARC) has identified other viruses and classified them as “well-established (Group 1) carcinogenic agents in humans”, and these include: hepatitis B virus (HBV), hepatitis C virus (HCV), high-risk human papillomavirus (HPV high-risk types), Epstein-Barr virus (EBV), human herpesvirus type 8 (HHV-8), and human T-cell lymphotropic virus type 1 (HTLV) [8]. 

The carcinogenicity of tumor virus follows two main pathways: 1. Direct, due to virus insertion and integration into the host DNA, resulting in alteration of gene structure and transcription [9]; and 2. Indirect, which involves chronic inflammation and immunosuppression arising from the infected cells [9,10]. 

As EBV modifies and influences the TME, it uses the indirect pathway of carcinogenesis to support malignant transformation in the host. Three of the mechanisms that EBV uses to transform the TME are explored in this paper, including overexpression of *IDO1*, synergism between *H. pylori* and EBV co-infection, and M1 to M2 macrophage switch. 

## 3. Direct Carcinogenesis: EBV Infectious Cycle and Related Oncogenic Alterations 

EBV has envelope glycoproteins that facilitate fusion with B-lymphocytes and epithelial cells. The glycoproteins that enable this entry process are gp350, gH, gL, gB and gp42 in B-lymphocytes and BMFR2, gH, gL and gB in epithelial cells [2]. After EBV enters B cells and epithelial cells, the viral genome is integrated into the host DNA and uses the host’s machinery to replicate during its lytic phase [11]. This primary infection may manifest asymptomatically or as symptomatic mononucleosis [12]. 

Then, EBV undergoes methylation, establishing latency in memory B cells. In this period of incubation, the virus does not replicate but can still alter the host’s gene expression and cellular signaling pathways [13]. Latent infection can present in several latency-forms, each expressing different lytic proteins that play a role in malignant transformation [14]. EBV-latency proteins contribute to creating a microenvironment that supports the transition of infected cells to cancer by suppressing the host’s antiviral immune defenses. 

Despite the ubiquity of EBV infection, only 1.8% of cancer deaths globally can be attributed to EBV-associated malignancies. Most immunocompetent hosts can control the infection and subsequent malignant transformation. The reasons some people develop a chronic EBV infection that can lead to malignancy could include immunodeficiency, genetic predisposition, and environmental factors [15]. 

EBV-mediated structural and epigenetic alterations as well as EBV-encoded gene products and miRNAs contribute to malignant transformation via immune escape [15]. 

### 3.1. EBV Cell-Cycle Dysregulation

EBV-encoded proteins and non-coding RNA play a role in the activation of cellular signaling pathways, including *NF-κB*, *PI3K/AKT, JAK/STAT, MAPK, TGF-β* and *Wnt/β*-catenin; the subsequent cell cycle dysregulation contributes to the development and progression of cancer [16]. 

In addition, viral gene products have been found to play a role in malignant transformation. For example, EBV-produced *LMP1* was found to hamper the mitotic G2 checkpoint, leading to chromosomal instability via the accumulation of somatic mutations after genotoxic stress, which plays a role in carcinogenesis [16,17]. Another set of EBV-latent gene products, *EBNA2* and *EBNA-LP*, were found in a recent study to alter the alternative splicing regulation of genes *NUMB* and *BCL-X*, which are involved in cell survival and proliferation after EBV infection [18]. Other EBV gene products that act as *BCL-2* homologs provide anti-apoptotic function and are also involved in oncogenesis [3]. 

### 3.2. EBV-Induced Epigenetic Mutations 

#### 3.2.1. DNA Methylation

EBV-induced DNA methylation affects the promoter region of approximately 886 genes involved in cancer related pathways [19]. Two of these genes, *PIK3CA* and *ARID1A*, are present with the highest methylation rate [20,21]. By hyper-methylating these genes, the tumor suppressor is inhibited, resulting in EBV-associated malignancies. In addition, proteins expressed by EBV during its latent period, like *LMP1* and *LMP2A*, may also directly methylate the promoter region of multiple genes and create abnormal epigenetic alterations in the host genome, resulting in EBV-malignancies [22]. 

EBV latent proteins can also reverse the silencing of genes involved in the lytic phase, which are normally hypermethylated. For example, there is a *BZLF1* gene product called Zta that reverses the silencing and enhances the expression of genes required for the lytic phase, and in doing so acts as a transcriptional activator [23,24]. 

#### 3.2.2. Histone Acetylation 

Histone acetylation makes chromatin more accessible, enabling gene transcription. For example, the viral genes *BZLF1* and *BRLF1* are tightly repressed during latency but can be activated via histone deacetylase inhibitors (HDACi) during viral lytic gene expression [25]. 

### 3.3. EBV miRNAs and Their Role in Immunosuppression 

In the EBV genome, there are two regions that contain more than 40 miRNAs. These short RNA segments regulate the expression of both viral and human genes [22]. Although non-coding, miRNAs play a role in antibody production, apoptosis, antigen presentation and recognition, communication between immune cells, and B cell transformation [26]. By regulating the immune landscape, miRNAs may directly and indirectly aid the infected host cell in malignant transformation. A recent meta-analysis evaluated the prognostic value of miRNAs in NPC and EBVaGC and found that miRNA expression is directly correlated with poorer prognosis [27]. 

## 4. Indirect Carcinogenesis: Tumor Microenvironment (TME) in EBV-Associated Malignancies 

The TME represents the ecosystem that encircles a tumor, and although its composition varies based on tumor type, it is often made up of immune cells, blood vessels, extracellular matrix cells, and resident cells. The TME reacts to the tumor by recruiting immune cells, but it can also be stimulated by the carcinogen to produce cytokines and chemokines that aid in malignant transformation [28]. To elucidate the critical role of the TME on tumor progression in EBV-malignancies, it is nearly impossible to make a xenograft of a primary EBV-malignancy; the tumor cells depend on their interaction with the TME to grow and proliferate [29]. 

EBV positive malignancies tend to harbor a TME that is highly infiltrated with immune cells (T cells, B cells, NK cells, dendritic cells, macrophages) and non-immune cells (fibroblasts and endothelial cells) in comparison to their EBV negative forms [29,30,31]. In EBVaGC, infiltrating CD8+ T cells are more abundant than CD4+ T cells with an approximate ratio of 10:1 [32]. Higher lymphocytes infiltration is also positively correlated with the presence of mature dendritic cells near cancer cells [33]. In comparison, B cells are present in lower numbers compared to T cells, and in EBVaGC they are more likely to localize in the stroma [32]. Other immunosuppressive components like cancer-associated fibroblasts and myeloid-derived suppressor cells have also been detected in EBVaGC [34,35]. EBVaGC is also characterized by increased *PD-L1* expression; current studies are investigating the safety and efficacy of durvalumab, an anti-PD-L1 agent in gastric cancer patients [36,37,38].

The mechanisms that EBV uses to transform the TME to its benefit are of clinical interest as they can be targeted to halt malignant progression [29,39]. 

## 5. EBV-Associated Gastric Cancer 

GLOBOCAN data from 2020 found that the global incidence of gastric cancer is 5.6%, with a mortality of 7.7% [40]. The different subtypes of gastric cancer can be classified based on their molecular and genetic features, which provides more information than the traditional histopathological classifications [41]. By classifying the subtypes of gastric cancer based on their genetic and epigenetic mutations, which are both drivers of carcinogenesis, we can develop novel therapeutics targeting molecular features that drive these changes [42]. Using a molecular classification system, The Cancer Genome Atlas (TCGA) classified the subtypes of gastric cancer into four categories: microsatellite unstable tumors (22%), genomically stable tumors (20%), tumor positive for Epstein–Barr virus (9%), and tumors with chromosomal instability (5%) [43]. 

Some studies suggest EBVaGC is more common in middle-aged males, typically found in the proximal stomach, and accompanied by a high lymphocytic infiltration [22]. Clinically, compared to gastric cancer subtypes that are not associated with EBV, the prognosis of EBVaGC is better and it has a lower rate of lymph node metastasis [44]. 

### EBVaGC TME

EBVaGC can be further classified into three histologic subtypes determined by the pattern of immune infiltration and specific TME components: lymphoepithelioma-like carcinoma (LELC), carcinoma with Crohn’s disease-like lymphoid reaction (CLR), and conventional adenocarcinoma (CA) [45]. LELC has the highest density of lymphocytic infiltration, followed by CLR and lastly CA. The intensity of the inflammatory response was correlated with better prognosis, elucidating the predictive value of the TME on clinical outcome [4]. 

Accordingly, establishing the mechanism that the TME uses to support the malignant transformation of EBV-infected cells may be helpful in developing new therapeutics. We proceed to present three mechanisms: *IDO1* overexpression, synergism between *H. pylori* and EBV during co-infection, and M1 to M2 pro-oncogenic transformation. 

## 6. EBV Induces IDO1, a Potent Immunosuppressor That Potentiates Malignant Transformation

Despite the trademark association between EBVaGC and a prominent lymphocytic reaction, there is a potent immune cell inhibitor enzyme that is often involved in the malignant transformation of this cancer. *IDO1* is the rate limiting enzyme needed to convert tryptophan (Trp) into kynurenine (Kyn) [46]. Many immune cells, including NK cells and cytotoxic T-lymphocytes, require Trp to proliferate and act against EBV-infected cells [47,48]. *IDO1* overexpression decreases the Trp/Kyn ratio, depleting immune cells of the Trp they require to function [46]. In multiple previous studies, *IDO1* expression has been associated with poor patient outcomes in not only gastric cancer, but also lung, prostate, esophagus, and uterine malignancies [46].

*IDO1* is induced by IFN-gamma, a cytokine that normally functions to activate the immune system but in the case of EBVaGC actually leads to *IDO1* overexpression, creating an immunosuppressive environment that encourages malignant transformation [49,50]. 

The clinical relevance of this lies in the development and evaluation of *IDO1* inhibitors and *IFNG*-pathway inhibitors in preventing malignant transformation and/or as potential therapeutic agents either alone or in combination with the standard of care. 

Recently, studies have shown clinical benefit of *IDO1* inhibitors in phase I and II clinical trials in melanoma, but not in phase III, delineating the need to identify cancer features that could predict treatment response [51]. Specifically, the *IDO1* inhibitor 1-methyl-tryptophan (1MT) is being used to evaluate its antitumor effectiveness when combined with the standard chemo-immunotherapy regimens [52]. However, a study in 2018 evaluating the effectiveness of epacadostat, an *IDO1* inhibitor, with Keytruda, a PD-1 inhibitor, in patients with unresectable or metastatic melanoma, showed no overall improvement as compared to Keytruda alone [53]. A list of clinical trials evaluating IDO1 inhibitors in cancers, either alone or in combination with other systemic therapies is shown in Table 1. As noted in this table, the *IDO* inhibitor indoximod, also known as 1-MT, is still undergoing clinical investigation in pediatric primary CNS tumors.

Notably, resistance to *IDO1* inhibitors could be attributable to the compensatory activation of the other two tryptophan catabolic enzymes *IDO2* and tryptophan 2,3-dioxygenase (*TDO*) [54]. Therefore, current studies are investigating pan *IDO1/IDO2/TDO* inhibitors to limit innate or acquired resistance to *IDO1* inhibitors and potentially limit adverse events with immunotherapy [55].

Some genes and other gene products specifically linked to the overexpression of the *IFN*-gamma pathway in EBVaGC include *STAT1*, *IFN* receptors, *IFN*-stimulated genes (ISGs) and IFN-regulatory factors (IRFs) [15], in addition to the EBV-encoded small RNAs and EBERs that modulate it [50]. Current efforts are attempting to address whether these genes and gene products can be targeted to downregulate the expression of *IFNG*, and hence reduce or prevent the progression of an immunosuppressive TME. 

## 7. Microbial Community as Part of the TME: EBV and *H. pylori* Co-Infection

In the stomach, multiple microorganisms coexist. Although *H. pylori* infection varies regionally, it is a ubiquitous bacterium, colonizing more than 50% of the global population [56]. About 2–3% of the people infected with *H. pylori* develop gastric carcinoma, making infection one of the strongest risk factors for this type of cancer [57]. Gastric cancer results when persistent infection by *H. pylori* leads to a chronic inflammatory response by the host, followed by metaplasia, dysplasia, and ultimately neoplasia. There are a myriad of host/bacterial/environmental interactions that play a role in potentiating this transformation [58]. One of these is the interaction between *H. pylori* and EBV in the TME, which synergizes malignant transformation. In fact, patients co-infected with EBV and *H. pylori* developed gastric cancer earlier in life than those without co-infection and were also noted to have more aggressive disease [59]. Some studies have hypothesized that the chronic inflammatory reaction in response to *H. pylori* recruits lymphocytes infected by EBV, enhancing the chances of their interaction with gastric epithelial cells [59]. 

**Figure 1:**
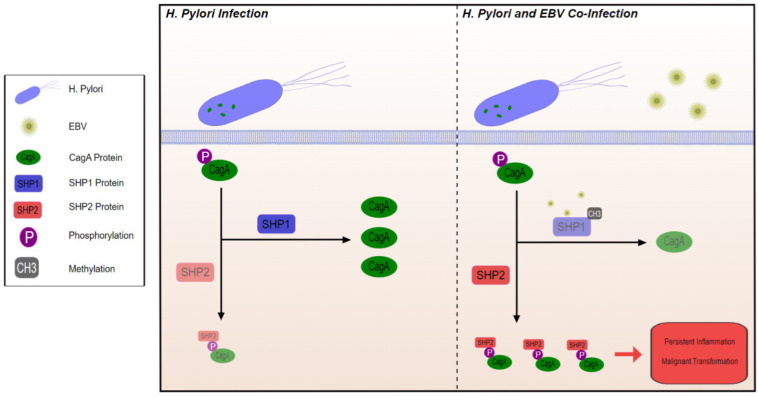
Through methylation and inhibition of SHP1, EBV co-infection with *H. Pylori* promotes interaction between phosphorylated CagA protein and SHP2 leading to inflammation and malignant transformation.

A different mechanism by which co-infection creates a TME that is more advantageous for malignant transformation involves CagA positive *H. pylori* strains. *H. pylori* strains include CagA positive and CagA negative strains. CagA positive strains produce the cagA protein, which becomes tyrosine phosphorylated in epithelial cells and subsequently interacts with *SHP2*, an oncoprotein that potentiates gastric cancer [60]. This interaction can be reduced by *SHP1*, which dephosphorylates CagA and hence minimizes its contact with *SHP2*. When a host is co-infected by *H. pylori* and EBV, EBV induces epigenetic silencing of *SHP1* via methylation [60], allowing tyrosine-phosphorylated CagA to interact with *SHP2* and thus potentiating malignant transformation (Figure 1) [61]. 

*H. pylori* eradication therapy is effective in preventing progression to gastric cancer, but data is lacking on whether *H. pylori* treatment can also prevent progression to cancer in hosts co-infected with EBV, and further studies to answer this question are needed [59]. 

*SHP2* inhibition is also of interest, either alone or in combination with the standard of care. Since *SHP2* is mostly a cytosolic protein, it cannot be targeted by antibodies. In addition, the therapeutic utility of *SHP2* inhibitors in this setting is also unknown and could present a therapeutic opportunity as there are currently about 10 *SHP2* inhibitor compounds in clinical development, mainly focusing on *KRAS* G12C mutated lung cancers and other solid tumors [62]. 

Other strategies, such as chimeric antigen receptor (CAR) T-cells have therapeutic potential and are being clinically studied [63]. Tabelecleucel, or tab-cel, is an off-the-shelf allogeneic EBV-specific T cell immunotherapy generated from healthy donors that is selected for each patient from a T cells library that is HLA-characterized using one EBV HLA restriction allele and at least one other matched HLA allele. It is in late-stage clinical development for EBV-positive post-transplant lymphoproliferative disease (EBV + PTLD) (NCT03394365), the ALLELE study, as well as other EBV-related diseases (NCT04554914) [64]. It has received Breakthrough Therapy Designation for EBV+ PTLD following allogeneic HCT by the Food and Drug Administration (FDA) and PRIME designation by European Medicines Agency (EMA) for the same indication. Tab-cel also has orphan drug designation in the U.S. and EU, and patients with EBV-related diseases are eligible to receive therapy via an ongoing expanded access program. 

## 8. Tumor-Associated Macrophages (TAMS), M1 to M2 Switch Mediated by TME

Macrophages are components of the innate immune system that primarily consist of two phenotypes: M1 and M2. Within the TME, classically activated macrophages (M1) are anti-tumorigenic: they create an inflammatory reaction (Th1) by producing inflammatory cytokines such as *IL-1β, IL-1α, IL-6, IL-12, IL-8, GFAP* and *TNFα* that ultimately lead to the destruction of tumor cells. In contrast, alternatively activated macrophages (M2) result in an anti-inflammatory reaction (Th2) by producing immunosuppressive cytokines such as *IL-4, IL-6, IL-10*, which promote pro-tumorigenic functions including angiogenesis and neovascularization [65]. 

Macrophages can be polarized, which means that they can switch their phenotype between M1 and M2 depending on the stimulatory factors that surround them. In the context of EBV, an increase in the M2 phenotype was observed during the latency phase [66]. The factors that mediate the switch in phenotype can be studied to elucidate potential therapeutics to augment the M1 phenotype and subsequently lessen the pro-oncogenic effects of M2. 

When tumors are under hypoxic stress, they recruit M2 via chemoattraction by endothelin-2 and vascular endothelial growth factor (*VEGF*) to use their angiogenic properties [67]. EBVaGC has been identified as a favorable predictive factor for treatment with *VEGFR2* monoclonal antibodies, specifically Ramucirumab [68]. Future clinical trials should evaluate the use of *VEGFR2* antibodies, such as bevacizumab, as a therapeutic modality in this tumor type. 

Another potent macrophage chemoattractant is *CCL2*, which interacts with its receptor, *CCR2* to recruit macrophages and stimulate their proliferation [69]. *CCR2* inhibitors have been shown to reverse M2 back to the M1 phenotype [70]. More data is needed to establish whether *CCR2* inhibitors, either as monotherapy or in combination, can effectively target EBV-related tumors. 

On the other hand, TAM receptors, including *Tyro3, Axl* and *MerTK*, are tyrosine kinases that stimulate polarization towards the M2 phenotype. These receptors can be inhibited to limit their pro-oncogenic effects. Oral inhibitors of *Tyro3, Axl* and *MerTK*, currently undergoing clinical testing, could also be evaluated in this setting [71]. 

The interaction between growth factor *CSF-1* and its receptor *CSF-1R* is another key pathway in the recruitment of macrophages and their polarization to M2 [69]. A phase I trial evaluating the effectiveness of emactuzumab, a monoclonal antibody against *CSF-1R*, was not successful in reducing malignant transformation either alone or in combination with paclitaxel. Although there was a reduction in M2 cells, this was not enough to derive anti-tumor benefit [72]. ABSK-021, which is undergoing first-in-human testing in solid tumors (NCT04192344), is an oral *CSF-1R* inhibitor which could be explored in this indication. Safety data of the single agent are expected later this year. Additionally, future studies should explore combinatorial approaches with *CSF-1R* inhibition and other systemic agents.

Finally, since macrophages share the same lineage with osteoclasts, the use of bisphosphonates is being evaluated to reduce macrophage polarization and reduce malignant transformation. For example, when zoledronic acid is phagocytized by M2 cells, it induces apoptosis and repolarization to M1 [72]. 

## 9. Perspectives and Conclusions

In summary, we reviewed three mechanisms by which EBV modifies the TME of the infected host for its benefit: *IDO1* overexpression, *H. pylori* and EBV co-infection, and M1 to M2 switch. We also explored how we can target these components of the TME and develop new potential treatment strategies. Many of the treatments described are still either in concept stage or in early phase exploration, so there are many questions that remain unanswered regarding their efficacy and tolerability that require further research. 

## Figures and Tables

**Table 1:** Clinical trials evaluating IDO1 inhibitors in cancers.

Trial Name	Identification	Phase	Status
Indoleamine 2,3-dioxygenase (IDO) Activity in Patients with Chronic Lymphocytic Leukemia (CLL)	NCT01397916	2	Completed
Indoleamine 2,3-Dioxygenase (IDO) Inhibitor in Advanced Solid Tumors	NCT02048709	1	Completed
NLG802 Indoleamine 2,3-Dioxygenase (IDO) Inhibitor in Advanced Solid Tumors	NCT03164603	1	Completed
Intraperitoneal Natural Killer Cells and INCB024360 for Recurrent Ovarian, Fallopian Tube, and Primary Peritoneal Cancer	NCT02118285	1	Completed
Epacadostat and Vaccine Therapy in Treating Patients with Stage III-IV Melanoma	NCT01961115	2	Completed
Pembrolizumab in Combination with Epacadostat or Placebo in Cisplatin-ineligible Urothelial Carcinoma (KEYNOTE-672/ECHO-307)	NCT03361865	3	Completed
Pembrolizumab + Epacadostat vs. Pembrolizumab + Placebo in Recurrent or Progressive Metastatic Urothelial Carcinoma	NCT03374488	3	Completed
Pembrolizumab Plus Epacadostat, Pembrolizumab Monotherapy, and the EXTREME Regimen in Recurrent or Metastatic Head and Neck Squamous Cell Carcinoma (KEYNOTE-669/ECHO-304)	NCT03358472	3	Active
Pembrolizumab Plus Epacadostat vs. Pembrolizumab Plus Placebo in Metastatic Non-Small Cell Lung Cancer (KEYNOTE-654-05/ECHO-305-05)	NCT03322540	2	Completed
Pembrolizumab (MK-3475) Plus Epacadostat vs. Standard of Care in mRCC (KEYNOTE-679/ECHO-302)	NCT03260894	3	Active
A Study of Pembrolizumab Plus Epacadostat with Platinum-based Chemotherapy versus Pembrolizumab Plus Platinum-based Chemotherapy Plus Placebo in Metastatic Non-Small Cell Lung Cancer (KEYNOTE-715-06/ECHO-306-06)	NCT03322566	2	Completed
Chemo-immunotherapy Using Ibrutinib Plus Indoximod for Patients with Pediatric Brain Cancer	NCT05106296	1	Recruiting
Pediatric Trial of Indoximod with Chemotherapy and Radiation for Relapsed Brain Tumors or Newly Diagnosed DIPG	NCT04049669	2	Recruiting

## Data Availability

Not applicable.

## Abbreviations

1-methyl-tryptophan	(1MT)
Alternatively activated macrophages	(M2)
Burkitt Lymphoma	(BL)
Carcinoma with Crohn’s disease-like lymphoid reaction	(CLR)
Chimeric antigen receptor	(CAR)
Classically activated macrophages	(M1)
Conventional adenocarcinoma	(CA)
EBV Related Breast Cancer	(EBVrBCa)
EBV-associated gastric cancer	(EBVaGC)
Epstein–Barr Virus	(EBV)
Hepatitis B virus	(HBV)
Hepatitis C virus	(HCV)
High risk human papillomavirus	(HPV high-risk types)
Histone deacetylase inhibitors	(HDACi)
Hodgkin’s Lymphoma	(HL)
Human herpesvirus type 8	(HHV-8)
Human T-cell lymphotropic virus type 1	(HTLV)
IFN-regulatory factors	(IRFs)
IFN-stimulated genes	(ISGs)
Indoleamine 2,3-dioxygenase	(IDO1)
Infectious mononucleosis	(IM)
International Agency for Research on Cancer	(IARC)
Kynurenine	(Kyn)
Lymphoepithelioma-like carcinoma	(LELC)
Nasopharyngeal Carcinoma	(NPC)
NK/T Cell Lymphoma	(NKTCL)
The Cancer Genome Atlas	(TCGA)
Tryptophan	(Trp)
Tumor microenvironment	(TME)
Vascular endothelial growth factor	(VEGF)
